# FLAN: feature-wise latent additive neural models for biological applications

**DOI:** 10.1093/bib/bbad056

**Published:** 2023-04-06

**Authors:** An-Phi Nguyen, Stefania Vasilaki, María Rodríguez Martínez

**Affiliations:** Department of Mathematics, ETH Zürich, Rämistrasse 101, 8092 Zürich, Switzerland; IBM Research Europe, Säumerstrasse 4, 8803 Rüschlikon, Zürich, Switzerland; Department of Mathematics, ETH Zürich, Rämistrasse 101, 8092 Zürich, Switzerland; IBM Research Europe, Säumerstrasse 4, 8803 Rüschlikon, Zürich, Switzerland; IBM Research Europe, Säumerstrasse 4, 8803 Rüschlikon, Zürich, Switzerland

**Keywords:** machine learning, deep learning, computational biology, interpretability

## Abstract

**Motivation:**

Interpretability has become a necessary feature for machine learning models deployed in critical scenarios, e.g. legal system, healthcare. In these situations, algorithmic decisions may have (potentially negative) long-lasting effects on the end-user affected by the decision. While deep learning models achieve impressive results, they often function as a black-box. Inspired by linear models, we propose a novel class of *structurally constrained* deep neural networks, which we call FLAN (Feature-wise Latent Additive Networks). Crucially, FLANs process each input feature *separately*, computing for each of them a representation in a common latent space. These feature-wise latent representations are then simply *summed*, and the aggregated representation is used for the prediction. These feature-wise representations allow a user to estimate the effect of each individual feature *independently* from the others, similarly to the way linear models are interpreted.

**Results:**

We demonstrate FLAN on a series of benchmark datasets in different biological domains. Our experiments show that FLAN achieves good performances even in complex datasets (e.g. TCR-epitope binding prediction), despite the structural constraint we imposed. On the other hand, this constraint enables us to interpret FLAN by deciphering its decision process, as well as obtaining biological insights (e.g. by identifying the marker genes of different cell populations). In supplementary experiments, we show similar performances also on non-biological datasets.

**Code and data availability:**

Code and example data are available at https://github.com/phineasng/flan_bio.

## INTRODUCTION

The recent success and fast development of deep learning models have fueled a parallel interest in *interpretable machine learning* research. Despite the universal approximation capabilities [1, 2] and generalization properties [3, 4] of deep learning, models often behave as black-boxes that do not reveal any information about the decision process. While not always necessary, interpretability is *critical* in applications with far-reaching impact, e.g. in legal practice and healthcare. In these high-stake scenarios, Rudin [5] advocates for *simple* and *already interpretable* models. Supporting this view, simple interpretable models perform similarly to more complex models in various real-world problems [6] and should therefore be preferred. Classical examples are decision trees [7], logistic regressors [8] or naive Bayes classifiers [9].

Unfortunately, transparent or *ante-hoc* models are not always accurate for high-dimensional and/or complex problems, where the representational capabilities of end-to-end deep learning models often result in improved performances. When these black-box models are used, interpretability can be achieved using *post-hoc* methods that aim to produce an explanation for a model that *has already been trained*. Examples of frequently used post-hoc methods are feature attribution methods [10] or example-based methods, such as MMD-Critic [11]. Recently, there has been an increased interest in developing *ante-hoc* deep learning models that attempt at retaining the representational and generalization properties of neural networks whilst still being interpretable [e.g. 12–14].

Contributing to this last class of interpretable models, in this paper, we introduce *FLAN* (Feature-wise Latent Additive Network). Inspired by linear models, FLAN computes a latent representation of each feature (or subsets thereof) *separately*. The latent representation of an input sample is obtained by *summing* the feature representations and then passed to a classifier network that performs the classification. We posit that these two constraints are what enable the interpretability of our model (Section 2.1).

We test FLAN on a diverse set of tasks in computational biology. FLAN achieves state-of-the-art performance (Section 3.1) in all tasks, and at the same time, it is able to provide insights into its decision process (Section 3.2). Our results are further corroborated by additional results outside the biological domain (Supplementary Section ‘Additional results: non-biological datasets’).

### Related work

Our work is motivated by interpretability. In the taxonomy [15] of interpretability methods, FLAN classifies primarily as a *local* method, since the model can be explained at individual sample points.

A simple way to inspect FLAN is via feature attributions. Notably, these feature importances can be computed natively from the model (Section 2.3.2), and there is no need to use post-hoc methods, such as gradient-based methods [10, 16–18], SHAP [19] or Relevance Aggregation [20], which would require more computational resources. This advantage is shared with Transformer models [21], where the attention scores can be interpreted as importances. However, the attention scores are a function of *all* the features, effectively modeling interactions. In Section 2.1, we argue that these explicitly modeled interactions decrease separability and, consequently, interpretability.

From a modeling perspective, FLAN belongs to the class of *ante-hoc structurally constrained* deep learning models. Established methods in this category are those that augment the network with prototype/prototypical-part-based reasoning, e.g. [12, 22]. A similar mechanism can also be achieved with FLAN (Section 2.3.3) without the need of an *ad-hoc* cost function or training strategy. Prototypes are similar in spirit to the concepts/feature basis used in Self-Explaining Neural Networks [SENN, 13]. Interestingly, Alvarez Melis and Jaakkola [13] also recognize the value of additivity/separability for interpretability. However, additivity is enforced only in the last layer of their model. The concepts (and their relevances) are computed as functions of the entire input space. Consequently, the interpretability with respect to the original input space is lost.

Closer in spirit to our model are the neural additive models [23], Explainable Boosting Machines [24] and the generalized additive models with interactions [25]. However, these previous works do not leverage a second prediction function after aggregating the per-feature functions, thus potentially losing the approximation capabilities of FLAN.

After presenting the details of the FLAN architecture, we will more thoroughly illustrate the core differences of FLAN compared with the mentioned constrained deep models (Section 2.4).

## METHODS

### Interpretability lessons from linear models

Linear models are considered prime examples of interpretable models, mainly due to two key characteristics:

- *Separability of features:* Linear models do not model *interactions* among features (unless an interaction term is explicitly added as an input feature). Hence, a user can examine the effect of each feature *separately* from the others, making the model easier to understand for a human user [26]
- *Predictability of the output:* Human users can easily understand the effect of a single feature on an output if the relationships are linear [27]. In the presence of multiple features, a human user can still easily predict their effect if the model is separable on each feature, as discussed in the previous point.

### Model architecture

Motivated by the previous section, we want to build a model for which the effect of single features can be predicted separately from each other. Let us assume that our goal is to learn a function $f: \mathcal{X} \rightarrow \mathcal{Y}$, where $\mathcal{X}$ has dimension $N$ and $\mathcal{Y}$ has dimension $M$. We propose to implement the function $f$ as 


(1) \begin{align*}& f(\mathbf{x}) = f(x_1, \dots, x_i, \dots, x_N) = \psi \Big( \sum_{i=1}^N \phi_i (x_i)\Big), \end{align*}

where:

-
$x_i$ $\mathrm{with} \; i=1,\dots ,N$ , are the features, i.e. the components of the input sample $\mathbf{x}$.
-
$\phi _i: \mathcal{X}_i \rightarrow \mathcal{Z}$ are feature functions that act separately on each individual feature $x_i$ and map them to the *same* latent space $\mathcal{Z}$ of dimension $D$. Note that the feature functions may either implement different functions or the same function for all features.
- The aggregate $\sum _{i=1}^N \phi _i (x_i) = \mathbf{z} \in \mathcal{Z}$ is the latent representation of the input sample $\mathbf{x}$.
- The predictor function $\psi : \mathcal{Z} \rightarrow \mathcal{Y}$ maps the sample latent representation to the output space $\mathcal{Y}$.

**Figure 1:**
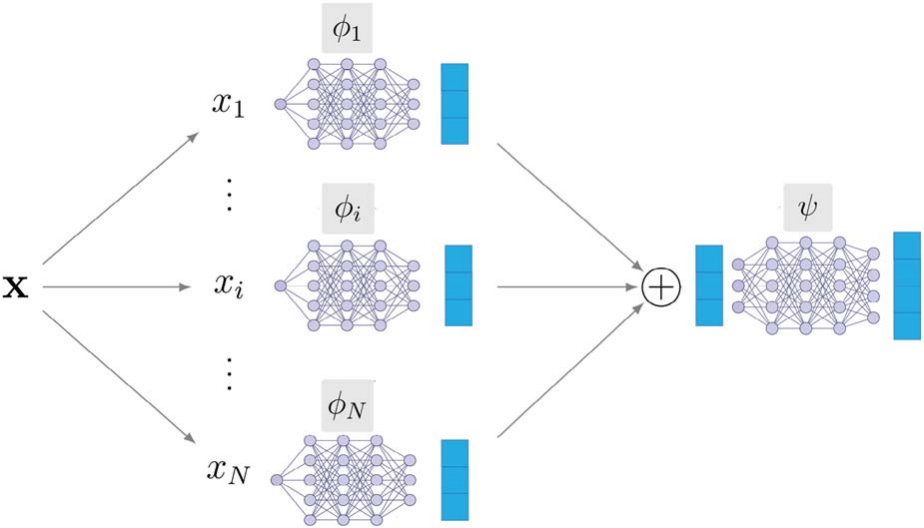
The base architecture of FLAN. A sample $\mathbf{x}$ is split in its features $x_i$. Each of the features is fed to a different function $\phi _i$ to compute the respective latent representations (blue rectangles). The processed features are next aggregated by summation and finally used as input of a predictive function $\psi$. In our work, the functions $\phi _i$ and $\psi$ are implemented as neural networks.

In this paper, we implement the feature functions $\phi _i$ and the prediction function $\psi$ as neural networks and learn them in an end-to-end fashion. Figure 1 provides a depiction of our model.

Before discussing the interpretability of our model (Section 2.3), a few additional remarks are useful.

**Universal approximation:** Since no interaction is explicitly modeled in Eq. (1), a concern that may be raised is if our model still retains the same approximation capabilities of traditional neural networks. The answer is given by the *Kolmogorov–Arnold representation theorem* [28], which informally states that any continuous function of a finite number of variables can be expressed in the form 


(2) \begin{align*}& f(\mathbf{x}) = f(x_1, \dots, x_N) = \sum_{q=0}^{2N} \Phi_q \Big(\sum_{i=1}^N \phi_{q,i} (x_i)\Big) \,. \end{align*}

Note that for interpretability purposes, we do not need the outer sum since it would separate neither the outputs nor the effects of the input features. We, therefore, use a more generic function $\psi$.

We finally remark that the Kolmogorov–Arnold theorem, essentially, states that multivariate functions can be expressed as iterated sums of univariate functions. In particular, this means that FLAN *does not dismiss feature interactions and simply leverages univariate functions to represent complex multivariate functions*. Rather, FLAN exploits an alternative function representation where feature interactions do not need to be explicitly modeled. Indeed, in Section 3.1, we show that FLAN *does* outperform univariate models (i.e. logistic regression). However, this comes at the price that the algorithmic interpretation is only *approximate* (Section 2.3.1).

**Feature subgroups:** In some applications, having a function applied to each individual feature may be detrimental. This is especially true when a single feature has no particular meaning. For example, in computer vision tasks, human users rarely understand image classification in terms of single pixels, but rather, in terms of higher-level concepts. Equation (1) can be adapted to consider features in *non-overlapping groups* instead of individually. Building on the computer vision example, an image could be processed in *patches* rather than single pixels.

**Structural information:** Some data types carry additional structural information, e.g. natural language or images. Equation (1) can be applied directly to these domains; however, for the purpose of parameter sharing, it may be useful to make the dependency on the structure explicit 


(3) \begin{align*}& f(\mathbf{x}) = f(x_1, \dots, x_N) = \psi \Big( \sum_{i=1}^N \phi (x_i; \theta, p_i)\Big) \,, \end{align*}

where $\theta$ are parameters common to all the feature functions, and $p_i$ encode the structural information. This is the same idea behind the positional embeddings used in transformer architectures [21].

### Interpreting FLAN

In this section, we will discuss the three main modalities to interpret FLAN. Concrete examples will then be presented in Section 3.2.

#### Algorithmic interpretation

The interpretability of FLAN stems from the fact that different features are processed *separately*. That is, we can analyze how each feature contributes to the prediction without being concerned about interactions.

A user can study the effect of a single feature $x_i$ simply by performing a prediction on that feature, i.e. $\psi (\mathbf{z}_i)$ with $\mathbf{z}_i = \phi _i(x_i)$. Note however that, since we are not making any assumption on the function $\psi$, the effect is not generally additive. In fact, assume that $\mathcal{Y}$ and $\mathcal{Z}$ are equipped, respectively, with norms $||\cdot ||_{\mathcal{Y}}$ and $||\cdot ||_{\mathcal{Z}}$. Furthermore, let us informally assume that we can compute the Taylor expansion of $\psi$ in a ‘large enough’ neighborhood of $\mathbf{z}_{*} = \sum _{j=1, \; j\neq i}^N \mathbf{z}_j$. Then, we have 


(4) \begin{align*}& \begin{split} ||\underbrace{\psi(\mathbf{z}_{*} + \mathbf{z}_i) - \psi(\mathbf{z}_{*})}_{(\Delta)} - \psi(\mathbf{z}_i)||_{\mathcal{Y}} & = \\ ||\mathbf{J}_{\mathbf{z}_*}\mathbf{z}_i - \psi(\mathbf{z}_i) + o(||\mathbf{z}_{i}||_{\mathcal{Z}})||_{\mathcal{Y}}, \end{split} \end{align*}

where:

-
$\psi (\mathbf{z}_{*} + \mathbf{z}_i)- \psi (\mathbf{z}_{*})$ is the change in prediction given by the additional information contained in the $i$-th feature;
-
$\mathbf{J}_{\mathbf{z}_*}$ is the Jacobian of $\psi$ computed at point $\mathbf{z}_{*}$;
-
$o(||\mathbf{z}_{i}||_{\mathcal{Z}})$ is the remainder term in the first-order Taylor expansion.

The right-hand side of Eq. (4) gets closer to zero the closer to linear $\psi$ becomes. Therefore, Eq. (4) is telling us that we can estimate the change in prediction $(\Delta )$ given by feature $i$ by looking at $\psi (\mathbf{z}_i)$, i.e. the prediction of that feature. The accuracy of the estimation will depend on how non-linear is $\psi$. Although, we are not making any assumption on $\psi$; in Section 3.2, we show that in practice, we can still estimate the effect of single features on the prediction.

#### Feature importance

In the previous section, we showed how to interpret FLAN in a way similar to linear models. However, FLAN can also be interpreted via feature importance, i.e. by computing a score *indicative* of how much a feature may influence the model’s prediction. Importantly, this can be done *natively* in FLAN, i.e. without the need for *post-hoc* methods, such as SHAP [19]. To see this, remember that since sample representations are the *sum* of individual feature representations, a feature $i$ that is mapped to a small vector $||\mathbf{z}_i||_{\mathcal{Z}}\approx 0$ will bring *no contribution* to the prediction. In contrast, the features with the highest norms are the most informative, i.e. they are useful for summarizing the original input. This means that we can use the norms of the feature latent representations as indicative of feature importance.

#### Example-based

Another typical way to interpret models is by finding prediction prototypes. That is, given a sample, an understanding of the model can be achieved by retrieving some other samples that the model predicts in the same way [11]. In FLAN, this type of interpretability can be achieved by looking at the nearest neighbors in the latent space $\mathcal{Z}$: since $\psi$ in our model is a neural network and, therefore, a Lipschitz continuous function, i.e. $||\psi ({\hat{\mathbf z}}) - \psi ({\tilde{\mathbf z}})||_{\mathcal{Y}} \leq L ||{\hat{\mathbf z}} - {\tilde{\mathbf z}}||_{\mathcal{Z}}$, the predictions performed on two samples with similar latent representations will be similar.

**Why different modalities?** When interpretability is needed in the context of machine-solved predictive tasks, there is potentially a *wide range* of possible parties interested in achieving an understanding of how the ML model works. These so-called ‘stakeholders’ may have distinct technical backgrounds and different motives for interpreting the model [29]. This in practice means that different stakeholders will prefer to interpret the same ML model in different ways [30]. We, therefore, argue that a model that can be natively interpreted in several different ways holds an advantage against other more limited models.

### Comparison to other constrained deep models

In Section 1.1, we positioned FLAN in the class of structurally constrained deep models. In this section, we elucidate the difference between FLAN and the cited constrained deep model.

**Neural additive models** [23]: neural additive models (NAMs) are the closest in formulation to FLANs. The main difference is that NAMs do not make use of a second function $\psi$, which degrades their approximation power. On the other hand, this allows a more precise algorithmic interpretability since there is no need of the same approximating assumption made in Section 2.3.1.

**SENNs** [13]: Self-Explaining Neural Networks also implement interpretability in terms of additivity. However, this additivity is enforced only in the last layer. Briefly, an SENN architecture is of the form $f(\mathbf{x}) = \theta (\mathbf{x})^T h(\mathbf{x})$, where $h(\mathbf{x})$ is a concept encoder neural network, and $\theta (\mathbf{x})$ is a relevance parametrizer neural network. Both networks output to a space of dimension $d$. Essentially, each of the $d$ elements of $h(\mathbf{x})$ encodes how much of a ‘concept’ is present in $\mathbf{x}$, while the corresponding element in $\theta (\mathbf{x})$ encodes the importance of that concept towards the prediction. Note that, in general, both networks take the whole input $\mathbf{x}$ as argument. This means that the prediction is separable in terms of the concept, but not in terms of the original input space, which we have argued is important for interpretability. Furthermore, SENNs require an extra post-hoc analysis step to understand the encoded concepts, while in FLANs, the features can be inspected, either individually or in subgroups (to form ‘concepts’), directly by means of the algorithmic interpretation (Section 2.3.1).

**ProtoPNet** [12, 23]: This class of prototype-based networks is similar to SENNs in the fact that the final prediction is obtained via a linear classifier $f(\mathbf{x}) = \mathbf{W} h(\mathbf{x})$. $h(\mathbf{x})$ keeps the role of a prototype/concept encoder, but the ‘concept importances’ are now input-independent. However, by leveraging the structure of CNNs, $h(\mathbf{x})$ is now separable in terms of the original input features. Furthermore, by up-sampling the output of the convolutional layers, the network can achieve an algorithmic interpretation similar to FLANs. The main drawback is that ProtoPNet has to be trained in a multi-stage process.

A performance comparison of FLAN against these methods in traditional (non-biological) benchmarks is reported in the Supplementary.

## RESULTS

### Classification performance

We report results for three exemplary biological and medical datasets. In all cases, we report the Area Under the Receiver Operating Characteristic Curve (ROC-AUC). FLAN performs on par with state-of-the-art established models. This holds true also on non-medical datasets (Supplementary Section ‘Additional results: non-biological datasets’).

#### Tabular data: single cell classification

We test FLAN on the task of classifying cellular populations in a single-cell RNA-seq dataset. The dataset consists of 40 000 fresh peripheral blood mono-nuclear cells from a healthy donor, where measurements of $32\,738$ genes were obtained by Zheng et al. [31]. Following their methodology, we select the 100 most variable genes, as well as additional *marker genes*. Our final gene set consists of 117 genes. We utilize the labels provided by the authors to classify the cells into 7 classes. The classes are highly imbalanced; therefore, we use SMOTE [32] for oversampling. This oversampling procedure has some risk of producing ‘unnatural’ samples, negatively biasing the trained model towards non-biologically plausible rules. However, we will see in Section 3.2 that, thanks to the interpretability of FLAN, this is not the case.

We train an FLAN where each gene is mapped by a *different* feature network $\phi _i$ to a 24-dimensional latent space. For performance evaluation, we compare FLAN to logistic regression and KNN classifiers with 7, 13 and 21 neighbors.

This task is relatively simple, and all tested models achieve good performance, as seen in Table 1. The logistic regressor, a linear model, achieves about 98% ROC-AUC. Both non-linear models, KNN and FLAN, provide a boost in performance, with FLAN outperforming KNN. Nonetheless, we will see that FLAN can be interpreted more similarly to a linear model (Section 3.2.1).

**Table 1:** ROC-AUC (mean and *standard deviation* over 10 runs) scores for the single cell classification task. FLAN (ROAR) denotes an FLAN model trained only on the markers shown in Figure 2 to further corroborate our biological interpretation (Section 3.2.1)

	ROC-AUC
Logistic regression	0.982 $\pm$*1e−16*
KNN-7	0.992
KNN-13	0.996
KNN-21	0.995
SVM	0.998 $\pm$*4e−7*
FLAN	0.997 $\pm$*0.0002*
FLAN (ROAR)	0.990 $\pm$*0.0007*

#### Text data: T cell receptor binding prediction

Next, we train FLAN to predict the binding between human T cell receptor (TCR) and epitope amino acid sequences. We use the same dataset used by Weber et al. [33] to train the TITAN model. This dataset is a combination of the VDJ database [34] and COVID-19 data acquired from the ImmuneRACE project [35]. It consists of $46\,290$ TCR beta chains associated with $192$ different epitopes. The sequences of variable lengths are homogenized by padding them with <PAD> tokens. Furthermore, the start and end of the sequences are marked with <START> and <STOP> tokens, respectively. The final lengths for TCRs and epitopes are 150 and 50, respectively. The authors provide two splits for evaluation. In the first split, referred to as *TCR split*, the train and test sets contain different TCR sequences, but epitopes are shared across splits. The second split, referred to as *strict split*, contains different TCRs and epitopes in the train and test sets. The TCR split can be used to investigate a model’s generalization to new TCRs, while the strict split is used to test generalization to unseen TCR-epitope pairs. Generalizing to new epitopes is a far more challenging task, due to the limited number of epitopes in the dataset.

We train an FLAN model that separately maps each amino acid into a latent space of dimension 128. We use the same feature network for all the amino acids, but we account for their position in the sequences by using a positional embedding, similar to those used in transformer models [21]. As baselines, we use TITAN, logistic regression and a Levenshtein-based KNN classifier.

Table 2 shows that FLAN outperforms the baseline KNN models by a large margin and achieves comparable results to TITAN [33], a state-of-the-art deep model for the task. In the TCR split, TITAN’s pretraining (TITAN fine-tuned) and augmentation strategies (TITAN f. semi. aug.) enhanced the accuracy by 2.6%, suggesting that a similar approach might benefit FLAN as well.

**Table 2:** ROC-AUC scores (10-fold cross-validation mean) on the TCR and strict splits. In the TCR split, the models are required to predict the specificity of unseen TCRs. In the strict split, the models need to generalize to both unseen TCRs and epitopes

	TCR split	Strict split
Logistic regression	0.749	0.505
KNN-7	0.824	0.53
KNN-13	0.819	0.533
KNN-21	0.81	0.533
TITAN fine-tuned	0.841	0.564
TITAN f. semi. aug.	0.867	0.564
FLAN	0.855 $\pm$ 0.006	0.558 $\pm$ 0.008

On the strict split, all models perform close to random. As mentioned before, this is an extremely challenging task due to the low number of epitopes available in the dataset.

#### Image data: Skin disease classification

We test FLAN by classifying a large collection of dermatoscopy images into 7 different diseases. We use the DermaMNIST dataset published by Yang et al. [36], which consists of 10 015 dermatoscopy 3-channels images resized to a 28$\times$28 pixel size. We further split the images into 49 non-overlapping patches of dimension $4x4$. The patches constitute the features that are separately processed by FLAN’s feature network. Similarly to the TCR-epitope binding task, the features are processed by the same feature network together with their positional information. The features are mapped to a 256-dimensional latent space. We compare FLAN to well-established vision models, including ResNets [37] and models obtained by AutoML frameworks [38–40], i.e. models whose architecture (such as type of layers, number of layers, type of connections, etc.) are automatically optimized.

FLAN achieves better performance compared with well-established methods, such as ResNets [37], in the classification dermatoscopy images (Table 3). However, a more automated architecture optimization could further improve the accuracy of our model, as suggested by the results obtained by AutoML frameworks.

**Table 3:** ROC-AUC scores in the skin lesion image classification task. Results for ResNet and AutoML models are reported by Yang et al. [36]. For FLAN, we report mean and *standard deviation* over 10 runs

	ROC-AUC
ResNet-18 (28)	0.899
ResNet-18 (224)	0.896
ResNet-50 (28)	0.886
ResNet-50 (224)	0.895
auto-sklearn	0.906
AutoKeras	0.921
Google AutoML Vision	0.925
FLAN	0.901 $\pm$*0.013*

### Interpretability analysis

In this section, we discuss the interpretability insights that can be extracted from FLAN using the 3 examples introduced in Section 3.1.

#### Single cell classification

The first way to interpret FLAN predictions is by looking at the feature importances (Section 2.3.2). Feature importances are usually analyzed in a *local* fashion, i.e. separately for each sample, and this is the strategy we will exploit later for the image task (Section 3.2.3). In tabular datasets, however, it is often convenient to have a more *global* view by mean-aggregating the importances computed on the whole dataset. For the single-cell classification task, this means that we compute the mean importance scores for all cells in the same class. In the following, we show that using simple aggregation, we obtain biologically corroborated conclusions about the knowledge learned by our FLAN model. However, in different tasks, more sophisticated aggregation methods may be required (e.g. Relevance Aggregation [21]).

**Figure 2:**
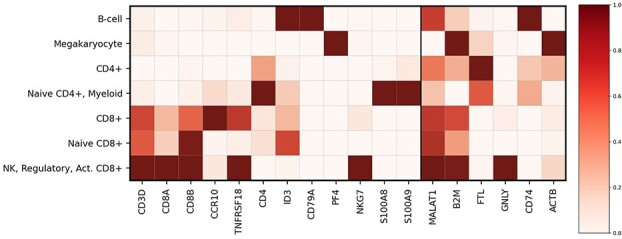
FLAN importance scores for the single cell classification task. Shown are the importance scores averaged over the training set for the 12 marker genes (the 12 leftmost genes on the $x$-axis) and for the 6 most variable genes over the whole dataset (the 6 right-most genes on the $x$-axis). The $y$-axis shows the 7 classes provided by Zheng et al. [31] translated to immune populations based on their marker genes expression. Weights are normalized for each gene. Darker colors indicate higher importance.

Figure 2 shows the aggregated importances. To simplify the visualization, we only show 18 genes on the $x$-axis, including the 12 markers used by Zheng et al. [31] and the 6 most variable genes out of the remaining 100 genes (Section 3.1.1). For comparison, Supplementary Figure 7 shows the importances for the same 12 markers and the 6 *most expressed* genes.

Our interpretability results, shown in Figure 2, corroborate the biological interpretation provided by Zheng et al. [31]. *CD8A* and *CD8B* genes encode the alpha and beta chains of the CD8 receptor [41] and are assigned high importances in all clusters containing CD8+ T cells (e.g. CD8+, naive CD8+ and NK+other classes). Furthermore, FLAN highlights ID3, which is critical in maintaining a naive state [42] in naive CD8+ cells. Similarly, CD4+ cells are associated with *CD4* and *ID3* markers, although the importance scores are weaker. *CD3D* encodes the delta subunit of the CD3 complex [43] and it is highly expressed in all T cell subpopulations. While *CD3D* seems to play an important role in differentiating CD8+ cells, FLAN fails to identify its importance in the CD4+ and naive CD4+ classes. This might be a consequence of the dataset imbalance: class CD4+ contains only 972 samples, whereas class CD8+ has 11785 samples. Another possible explanation might be the weak signal characterizing *CD4*, *CD3D* and *ID3* in CD4+ and naive CD4+ cells in the dataset (Supplementary Table 8). Without incorporating prior biological knowledge, it is expected that FLAN will miss established biological markers if they are weakly expressed in the dataset, as is the case of *CD4*, *CD3D* and *ID3*. As expected, B cells are associated with *CD79A* [44], while megakaryocytes with *PF4* [31, 45]. The class *NK (natural killer)+other* is a mixture of different cells, resulting in many marker genes playing an important role. The presence of *CD8A* and *CD8B*, in addition to *NKG7*, suggests that it likely consists of activated T cells [46], while *TNFRSF18* indicates the presence of regulatory T cells [47]. Moreover, the importance attributed to *TNFRSF18* and *NKG7* confirms that NK cells are part of this aggregated class [31, 48]. Lastly, the enrichment of *S100A8* and *S100A9* in the naive CD4+ class implies the presence of myeloid cells [49]. We validate our findings by retraining an FLAN model using *only* the features shown in Figure 2. This evaluation strategy is known as *RemOve-And-Retrain (ROAR)* [50] and assesses if the detected features are actually important, in the sense of being informative for the task. As reported in Table 1 [row ‘FLAN (ROAR)’], this is indeed the case since the drop in performance is negligible.

After this global overview of the model, we can achieve a more specific insight on the decision process for a single sample via algorithmic interpretation of our model (Section 2.3.1). Table 4 exemplifies such interpretation modality for a sample that our trained FLAN classifies as a CD8+ cell. The table shows the top 3 classes (row ‘Full’) predicted by FLAN for the sample using the expression of all 108 available genes. We then analyzed FLAN predictions when only the value of an individual gene is provided. Table 4 shows the predictions obtained using individually the 7 most important genes for this sample, as scored by the latent feature norms. These results show that each of the most important genes provides strong evidence towards CD8+ cells. The top 4 genes further provide strong evidence for the NK+other class, which may explain why this is the second most probable class for this sample. Finally, ACTB and CD8B seem to contribute evidence towards a naive state for the cell, explaining the third most probable class for the sample. It is interesting to note that the global overview given by the aggregated importances does not necessarily reflect what happens at the individual sample level. For example, NKG7 does not play an important role in general for class CD8+ in Figure 2; however, it significantly contributes to the classification of the sample reported in Table 4.

**Table 4:** Algorithmic interpretation of the single cell classification task. As an example, we show a sample classified by FLAN as a CD8+ cell. The top 3 predicted classes for the sample are shown in the row Full. In the other rows, we report the 7 most important genes for the sample (as scored by the latent representation norm) together with the top 3 predicted classes *if FLAN only had access to that gene for the classification*. The numbers in parentheses indicate the probability of being classified in each class

	Predicted class: CD8+ cell		
Full	*CD8+* (0.3)	*NK+oth* (0.11)	*NaiveCD8+* (0.11)
MALAT1	*NK+oth* (0.62)	*CD8+* (0.24)	*N.CD4+Myel* (0.04)
B2M	*NK+oth* (0.47)	*CD8+* (0.34)	*CD4+* (0.08)
CCL5	*CD8+* (0.31)	*NK+oth* (0.15)	*Megakar* (0.15)
NKG7	*CD8+* (0.42)	*NK+oth* (0.39)	*B-cell* (0.04)
ACTB	*CD8+* (0.31)	*NaiveCD8+* (0.26)	*CD4+* (0.11)
CD8A	*CD8+* (0.24)	*NK+oth* (0.2)	*N.CD4+Myel* (0.13)
CD8B	*CD8+* (0.31)	*NaiveCD8+* (0.19)	*B-cell* (0.12)

To compute example-based explanations, we split the training samples into 7 groups based on their *predicted* classes and use K-Medoids [51] to select 6 prototypes for each group. Supplementary Figures 8–14 display the gene expression of the prototypes for the 7 groups. These example-based explanations give an overview of the typical gene expression patterns for each of the predicted classes.

#### TCR binding

For textual datasets, it is less meaningful to visualize a global aggregate of importances. We, therefore, focus on single samples in our analysis.

**Figure 3:**
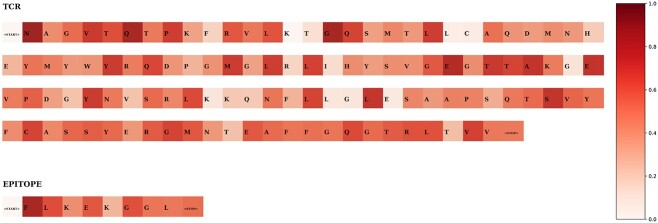
FLAN importance scores for the TCR-epitope binding prediction task. We report scores for a sample classified as binding. For better visualization, we omit the <PAD> tokens since they are assigned low scores. Darker colors mean higher importance.

Figure 3 shows the individual amino acids importances for both the TCR and the epitope sequences for a sample predicted as binding. Reassuringly, FLAN assigns low importance to the ‘<PAD>’ (not reported in the figure for better visualization) and ‘<START>’ positions, and moderate to high importance to the amino acids in the epitope and TCR sequence. This is reasonable, as the ‘<PAD>’ and ‘<START>’ do not contain meaningful biological information. However, the ‘<STOP>’ position shows a moderate weight. This might indicate that FLAN either tracks the length of the sequences or misunderstands the stop indicator as a segment of the sequence.

An analysis of the most important amino acids (Supplementary Figure 15) revealed that the epitope’s amino acids are ranked more frequently among the top 10 (i.e. the ones with the highest norms). Moreover, it does not appear to be a preference towards particular amino acids in either of the sequences (e.g. the most picked amino acid for the TCR sequence is *V* with approximately 10% frequency).

In an attempt to simplify the interpretability of the model, we retrain FLAN by adding a regularizer in order to sparsify the feature importances. We observe a trade-off between FLAN’s performance and the feature importance sparsity (see Supplementary section B.5.1 and Figure 18).

A possible explanation could be that, from a biochemical point of view, a single amino acid is not sufficient to explain the binding between two proteins. A more accurate description might be achieved if we use $k$-mers of amino acids, i.e. segments of $k$ consecutive amino acids, as features. To test this hypothesis, we trained an FLAN using 3-mers and obtained a much sparser explanation at the price of a slightly decreased accuracy (Supplementary Section B.5.2). This might be because modeling amino acid sequences as lists of $k$-mers increase the number of possible input features that need to be learned (i.e. from 20 amino acids for $k=1$–$20^3$ possible triplets for $k=3$). Training such a model requires a larger number of samples, which might explain the slightly lower accuracy.

Similarly to the previous examples, we can interpret the model using the example-based modality. In Supplementary Figure 16, we show an example-based interpretation of this sample. More precisely, we show the original sample and its 3 closest neighbors, utilizing the latent representation of the samples and the euclidean distance.

**Figure 4:**
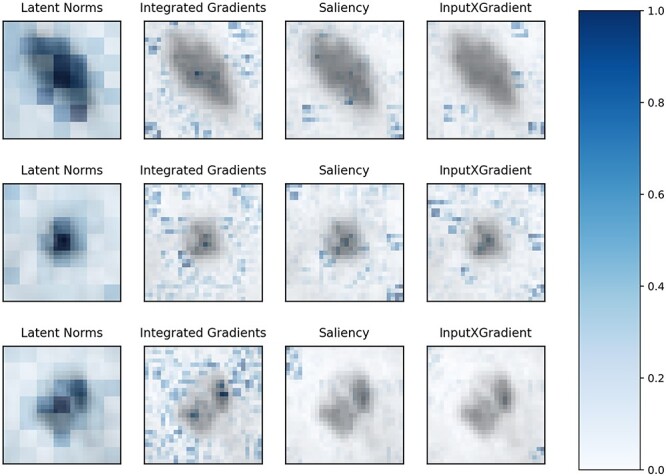
FLAN importance scores (leftmost column) for the skin lesion classification task. We compare the natively computed importance scores with 3 post-hoc methods. From the second leftmost column to the right: Integrated Gradients [18], Saliency [52] and InputXGradient [53]. Darker colors mean higher importance. We show the comparison for 3 different samples. As it is visually noticeable, FLAN identifies more accurately than the other 3 tested methods the skin lesions.

#### Skin disease classification

We begin by interpreting FLAN via feature importances, where the features are $4x4$ patches, as detailed in Section 3.1.3. The leftmost column of Figure 4 shows the importances computed as the norms of the latent representations of the image features for 3 different samples. As made evident in the figure, FLAN does a very good job at highlighting the skin lesion. We compare FLAN importances to scores computed using 3 widely post-hoc methods *applied to FLAN* [18, 52, 54]. The results are shown in the 3 rightmost columns in Figure 2, where we observe that, contrarily to FLAN, these 3 methods mostly highlight healthy skin areas as important regions. To evaluate the correctness of each method, we perform a quantitative test: we first identify the patches with the highest score for each method, and then, we remove them from the sample, i.e. we remove them from the feature summation step in FLAN and reclassify the image (Since the post-hoc methods provide pixel-level maps, to make them more comparable to FLAN, for each patch, we just pick the highest importance value within that patch). Supplementary Figure 17 shows that FLAN’s accuracy exhibits the highest drop when we exclude the patches ranked according to their latent representation norm rather than according to the attribution scores of the post-hoc methods. These results provide evidence that post-hoc methods do not reliably describe what our model is using for classification.

**Figure 5:**
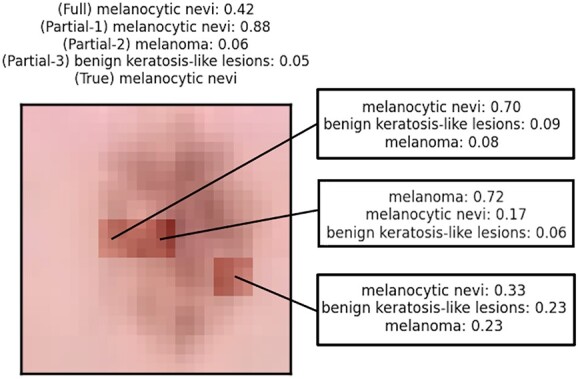
Algorithmic interpretation of FLAN on the skin lesion classification task. In the leftmost image, we show the reference sample that we are interpreting. We highlight the top 3 most important patches as scored by the latent norm representation. On top of the image, we report the top 3 predicted classes and their probabilities computed using only the 3 highlighted patches aggregated together (Partial-1,2,3). For reference, we provide also the prediction on the full image (Full) and the true label (True). In the 3 rightmost boxes, we show the top 3 predictions for the top 3 most important patches.

Figure 5 demonstrates the algorithmic interpretation of FLAN. The figure shows a sample correctly classified as *melanocytic nevi* together with the individual predictions of the top 3 patches (leftmost image). All three patches provide strong evidence for the class.

**Figure 6:**
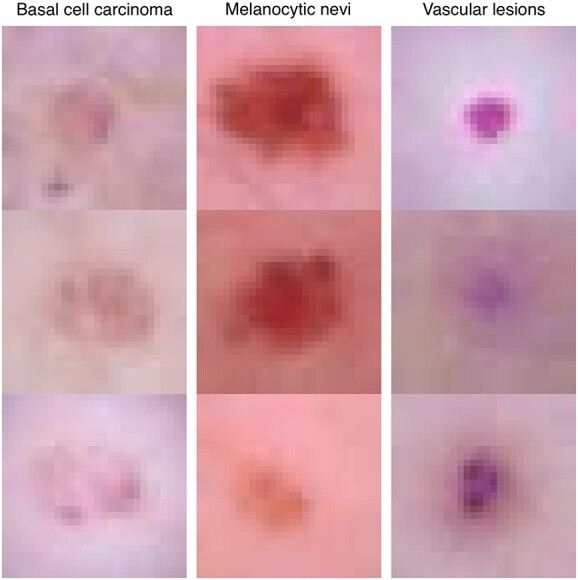
Example-based interpretation for the skin disease classification task. For 3 exemplary classes (columns), we show 3 prototypical samples that FLAN classifies in those classes.

Focusing last on interpretability by examples, Figure 6 displays typical prototypes for 3 different classes: *basal cell carcinoma*, *melanocytic nevi* and *vascular lesions*. Example-based explanations do not explicitly provide an algorithmic understanding of the model: it is up to the user to uncover the decision process of the model by looking at the prototypes. For example, looking at Figure 6, it seems that the rotation of the lesion and the contrast and brightness of the image do not play any role in the classification process. On the other hand, the color of either the healthy skin or the lesion might be important. For this particular case, the other explanation modalities have provided evidence supporting the conclusion that the lesion drives mainly the classification. In Supplement Section ‘Example-based interpretation for the image classification task’, we discuss more about the example-based interpretation of this task (Figures 20 and 21).

## DISCUSSION

In this paper, we introduced a class of *accurate and interpretable* models, which we call FLAN. In terms of prediction accuracy, our results show that our model can achieve state-of-the-art performance on biological datasets, as well as complex non-biological datasets (Supplementary Section ‘Additional results: non-biological datasets’). At the same time, we demonstrate how our model can be intrinsically interpreted *without the need for post-hoc methods*. Furthermore, our model can potentially be interpreted in different ways, therefore adapting to the needs of different users (Section 2.3).

This flexibility in terms of interpretability can be further increased by allowing the user to define what constitutes a feature. For example, in the TCR-epitope binding prediction task (Section 3.1.2), defining features as single amino acids results in dense explanations, likely because combinations of 2 or 3 amino acids are necessary to explain protein binding (Section 3.2.2). Therefore, to increase interpretability without excessive loss of performance, it may instead be necessary to define groups of 2 or more amino acids as inputs to the feature networks (Supplementary Section ‘FLAN with k-mers’).

There are multiple directions to explore for further development of our work. Similarly in spirit to using subgroups of features as inputs to feature networks, it would be interesting to explore a hierarchical version of FLAN. That is, an FLAN model with multiple steps of aggregation. For example, in the TCR-epitope task, each amino acid could be processed individually by a first set of feature networks. Then, the latent representations of each amino acid could be aggregated in small groups of $k$ amino acids, which would be further processed to obtain *protein domain* latent representations. Finally, these aggregated latent representations would be summed together and passed to the classifier. This approach could carry the advantage of providing hierarchical explanations, i.e. instead of simply highlighting individual amino acids as explanations, as FLAN currently does, we could find out which protein domains are more important for the binding, and within these domains, which amino acids play a more important role. Hierarchical explanations might increase the ability of FLAN to adapt to different stakeholders.

Our experiments suggest that FLAN could benefit from automated machine learning frameworks to optimize its architecture components. An interesting question to investigate is how automatic architecture optimization impacts FLAN interpretability, since some assumptions may be needed to effectively understand the model using certain modalities, e.g. algorithmic interpretation (Section 2.3.1). Along these lines, we further plan to more quantitatively evaluate the interpretability of FLAN via either user studies [55] or more programmatically defined metrics [56].

**Key Points**

- Ante-hoc interpretable/transparent models may not perform well on complex data where neural networks usually excel.
- Linear models are arguably interpretable for two main reasons: separability of the features and predictability of the input–output relationship.
- We design a constrained neural network model, FLAN, that can be interpreted *similarly* to linear models.
- We demonstrate how, despite the structural constraints, FLANs can still perform well on various tasks, both biological and non-biological.
- At the same time, thanks to these structural constraints, we can get an insight into the decision process of FLANs.

## Supplementary Material

FLAN_supplementary_bbad056

## Notes

**An-phi Nguyen** obtained his Bachelor’s degree at Politecnico di Milano and his Master’s degree at ETH Zürich in applied mathematics. After few years in startups, he started his PhD studies at IBM Research Europe, jointly with ETH Zürich. His research focuses on interpretable methods for machine learning, with applications in healthcare. His goal is to design trustworthy models that both assist clinicians during diagnosis, and can help unveil novel biological insights from various datasets.

**Stefania Vasilaki** completed her undergraduate studies in Mathematics at the National and Kapodistrian University of Athens. She completed her Master’s studies at ETH Zürich and was part of the Computational Systems Biology group at IBM Research Europe. She is currently a Machine learning Scientist at Roche, where she works on designing new antibodies for drug discovery using generative models and graph neural networks.

**María Rodríguez Martínez** did her undergraduate studies in Physical Sciences at Universidad Complutense de Madrid. She did her PhD in Theoretical Cosmology at the Institut d′Astrophysique de Paris. In 2007, she transitioned into the field of Systems Biology, first as a postdoc at the Weizmann Institute of Science in Rehovot (Israel), and next, at Columbia University (USA). Mariía joined IBM as a Research Staff Member in November 2013. Her research focuses on integrating multi-modal datasets to build comprehensive molecular models of cancer that can help clinicians to provide better diagnoses and suggest personalized therapies.
