# Supplementary material for: FLAN: feature-wise latent additive neural models for biological applications
Source: Brief Bioinform. 2023 Apr 6;24(3):bbad056. doi: 10.1093/bib/bbad056 (PMC10199769; doi:10.1093/bib/bbad056)
Supplement: FLAN_supplementary_bbad056 [file flan_supplementary_bbad056.pdf]

Summary of datasets

Table 5. Summary of biological datasets

Dataset	Dataset Type	# samples	Input Dimension	# classes	Short Description	References
Single Cell	Tabular	40000	117	7	Predict cell type from expression data	Zheng et al. [31]
TCR-Epitope	Text	46290	200	2	Predict binding of a TCR-epitope	Weber et al. [33]
DermaMNIST	Image	10015	32 x 32 x 3	7	Predict skin cancer type	Yang et al. [37]

Table 6. Summary of non-biological datasets

Dataset	Dataset Type	# samples	Input Dimension	# classes	Short Description	References
COMPAS	Tabular	7214	11	2	Predict recidivism risk	Larson et al. [58]
adult	Tabular	48842	14	2	Predict income from demographics data	Kohavi [59]
heart	Tabular	303	75	5	Predict risk class for heart disease	Detrano et al. [60]
mammo	Tabular	961	14	2	Predict cancer biopsy outcome	Elter et al. [61]
AGNews	Text	127600	75	4	Predict news topic from title	Zhang et al. [62]
IMDb	Text	50000	300	2	Predict movie reviews (pos vs. neg)	Maas et al. [63]
MNIST	Image	60000	28 x 28 x 1	10	Predict digits	LeCun et al. [64]
SVHN	Image	600000	32 x 32 x 3	10	Predict digits	Netzer et al. [65]
CUB	Image	11788	224 x 224 x 3	200	Predict bird species	Welinder et al. [66]

Additional results: biological datasets

Additional figures for the single cell classification task

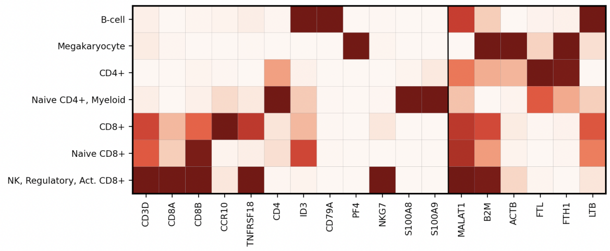

Fig. 7. FLAN importance scores for the cellular clustering task. Shown are the importance scores averaged over the training set for 12 marker genes and the 6 most expressed genes over the whole dataset. Weights are normalized for each gene.

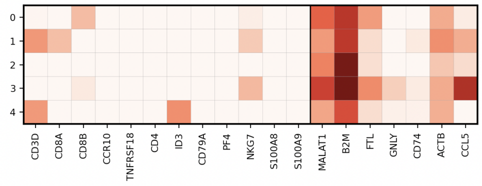

Fig. 8. Example-based explanation for the CD8+ cluster.

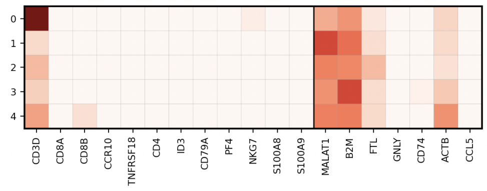

Fig. 9. Example-based explanation for the Naive CD8+ cluster.

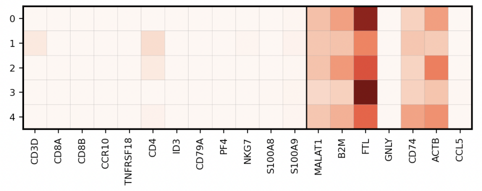

Fig. 10. Example-based explanation for the CD4+ cluster.

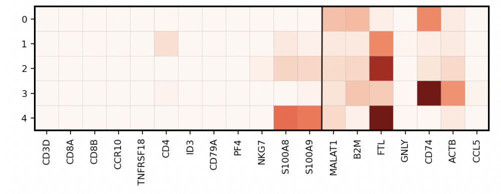

Fig. 11. Example-based explanation for the Naive CD4+, Myeloid cluster.

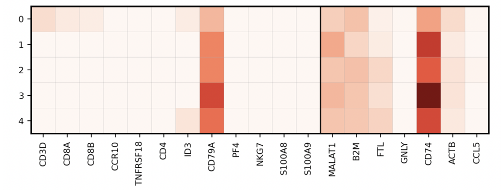

Fig. 12. Example-based explanation for the B cell cluster.

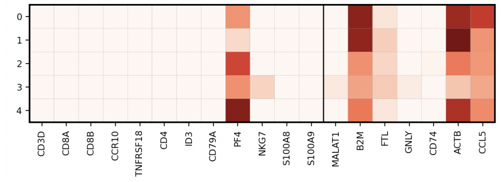

Fig. 13. Example-based explanation for the Megakaryocyte cluster.

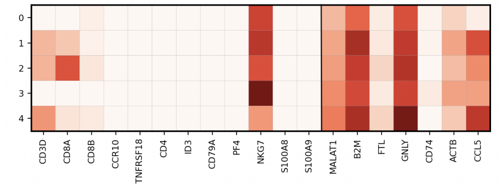

Fig. 14. Example-based explanation for the NK, Regulatory, Act. CD8+ cluster.

Additional figures for the TCR-epitope binding task

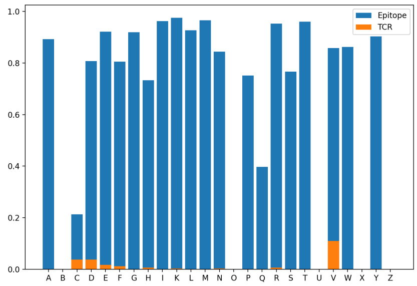

Fig. 15. FLAN's amino acid preference for the TCR and the epitope sequences. Each bar represents the frequency that the amino acid is ranked among the top 10. The majority of the top 10 amino acids comes from the epitope.

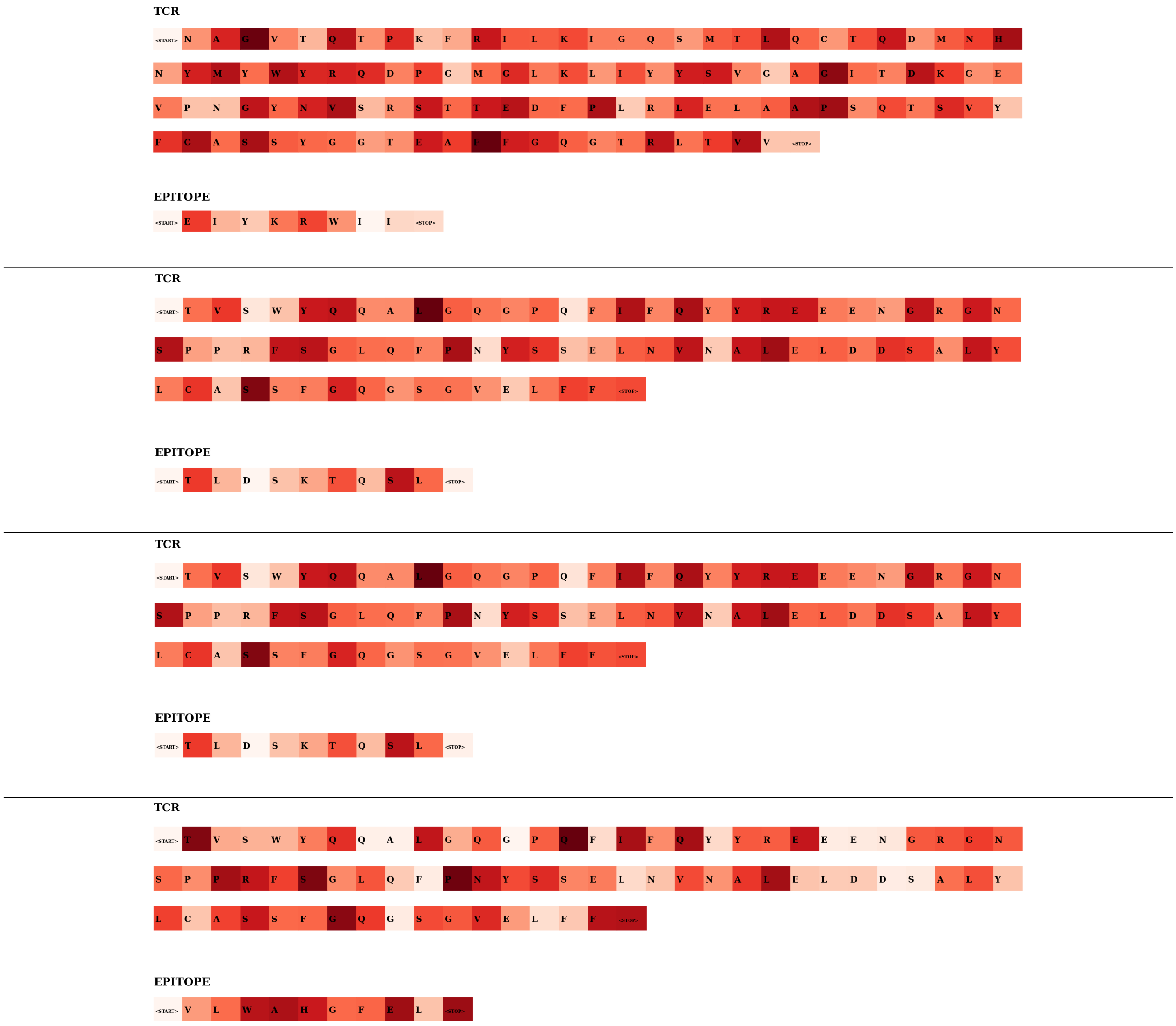

Fig. 16. Example-based interpretation of a sample in the TCR-epitope binding task. We show the original sample and its 3 closest neighbors, utilizing the latent representation of the samples and the euclidean distance.

Additional figures for the image classification task

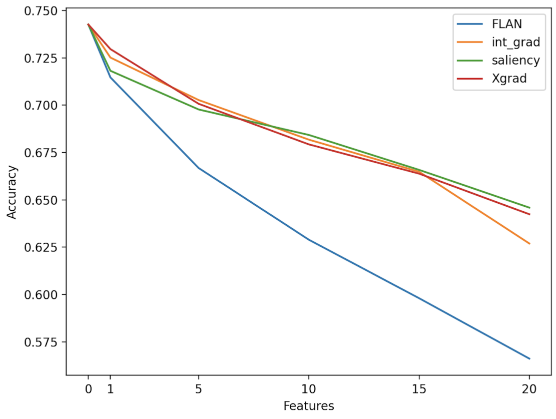

Fig. 17. Accuracy drop after excluding subsets of important features identified by FLAN, Integrated Gradients, Saliency and InputXGradient.

Marker genes for the single cell classification task

Table 7 displays the genes (referred to as *marker genes*) used by Zheng et al. [31] to cluster the cells into the immune subpopulations. Since there exist many marker genes that characterize immune populations, Table 7 does not represent the ground truth and is only utilized to identify the immune subpopulations in this task. Table 8 shows the mean expression of the marker genes for the 7 clusters. The mean gene expressions and FLAN's gene importances are highly correlated. Noticeably, *CD3D* is expressed 22 times less in the CD4+ cluster than in the CD8+ class and 7 times less in Naive CD4+ comparing to the Naive CD8+ cluster. This might explain why FLAN fails to assign *CD3D* a high importance score in the CD4+ and Naive CD4+ clusters. FLAN only detects the biological signals in our data, and it doesn't have access to prior biological knowledge. Similarly, the mean expression of *ID3* is less than 0.05% in the Naive CD4+ cluster, so FLAN might not consider *ID3* very important due to its weak signal.

Table 7. Important genes for identifying immune subpopulations based on Zheng et al. [31].

	Markers
B-cell	CD79A
NK	NKG7, TNFRSF18
Megakaryocyte	PF4
Myeloid	S100A8, S100A9
CD4+	CD3D, CD4
Naive CD4+	CD3D, CD4, ID3
Memory CD4+	CD3D, CD4, CCR10
Regulatory T cells	CD3D, TNFRSF18
CD8+	CD3D, CD8A, CD8B
Naive CD8+	CD3D, CD8A, CD8B, ID3
Memory CD8+	CD3D, CD8A, CD8B, CCR10
Activated CD8+	CD3D, CD8A, CD8B, NKG7

Table 8. Mean expression of the marker genes in the training dataset for the 7 clusters.

	CD8+	Megakaryocyte	CD4+	Naive CD4+, Myeloid	Naive CD8+	B-cell	NK, Regulatory
CD3D	1.560	0.062	0.072	0.192	1.394	0.068	1.336
CD8A	0.176	0.013	0.011	0.005	0.130	0.009	0.530
CD8B	0.246	0.000	0.019	0.027	0.363	0.012	0.413
CCR10	0.049	0.000	0.002	0.010	0.001	0.003	0.005
TNFRSF18	0.049	0.000	0.001	0.006	0.004	0.014	0.050
CD4	0.044	0.000	0.145	0.314	0.052	0.006	0.004
ID3	0.053	0.000	0.011	0.043	0.116	0.184	0.006
CD79A	0.017	0.000	0.011	0.028	0.039	3.663	0.010
PF4	0.005	20.114	0.002	0.039	0.003	0.000	0.004
NKG7	1.641	0.269	0.191	0.265	0.046	0.098	11.510
S100A8	0.010	0.000	0.210	7.920	0.011	0.015	0.012
S100A9	0.029	0.000	1.083	9.036	0.017	0.018	0.017
MALAT1	39.212	2.620	28.044	16.067	42.131	37.017	48.752
B2M	25.366	33.193	16.174	6.778	17.917	13.084	32.818
FTL	5.015	10.836	32.986	23.081	4.461	5.805	5.276
GNLY	1.362	0.355	0.128	0.412	0.071	0.144	16.827
CD74	0.861	0.183	7.159	9.359	0.387	23.318	1.061
ACTB	8.423	36.081	15.526	6.543	6.619	5.666	11.718
CCL5	3.315	12.092	0.118	0.180	0.239	0.137	8.766

Additional results for TCR-epitope binding task

Sparse FLAN models

To enforce sparsity to the feature importances we trained FLAN with additional penalties, namely l1, l2 penalties for the network's parameters and a custom penalty penalizing the sum of the feature norms. The sparsity of the model trained with the latter penalty is presented in figure 18. However, we observed a significant drop in the performance. In the TCR-split the ROC-AUC score dropped to 0,80 and the Balanced Accuracy dropped to 0,73. Our analysis showed that the more sparse the model becomes, the worst it performs. The trade-off between model accuracy and the sparsity, indicates that FLAN requires as much information (e.g. amino acids) as possible to predict sequence binding.

FLAN with k-mers

Similarly to learning features from non-overlapping patches in image-based tasks, we could utilize k-mers of amino acids to learn features that will summarize information from a neighborhood of size k. In this setting, we utilize a convolutional network with kernel size and stride equal to 3 to learn the features. Figure 19 displays a typical example of a binding pair. We observe more sparsity when using 3-mers, while the performance is still comparable to TITAN [33]. More specifically, the ROC-AUC score is 0,86 and 0,55 and the Balanced accuracy is 0,78 and 0,52 in the TCR and the strict split respectively.

Example-based interpretation for the image classification task

In this section, we discuss the third modality for interpreting FLAN, i.e. by examples. First, we separate the samples into 7 groups based on FLAN's predictions and perform K-Medoids with 3 clusters for each group. This results in 3 prototypes for each group, which can be used to identify the group's morphological characteristics. Figure 20 shows the results for the 7 groups. It seems that the rotation of the lesion and the contrast/brightness don't contribute much to the classification, while the color and the shape of the lesion may be important. For instance, the prototypes in the *Melanoma*, *Melanocytic nevi* and *Vascular lesions* classes are darker and with better defined shapes comparing to the rest. Another way to interpret FLAN with examples is to look for the nearest and the furthest neighbor in the latent space Z. Figure 21 displays the results for a sample predicted as *melanocytic nevi*. Its nearest neighbor is also classified as *melanocytic nevi*, and they share some morphological characteristics such as round shape and dark pink color. Its furthest neighbor has an irregular shape and a darker color.

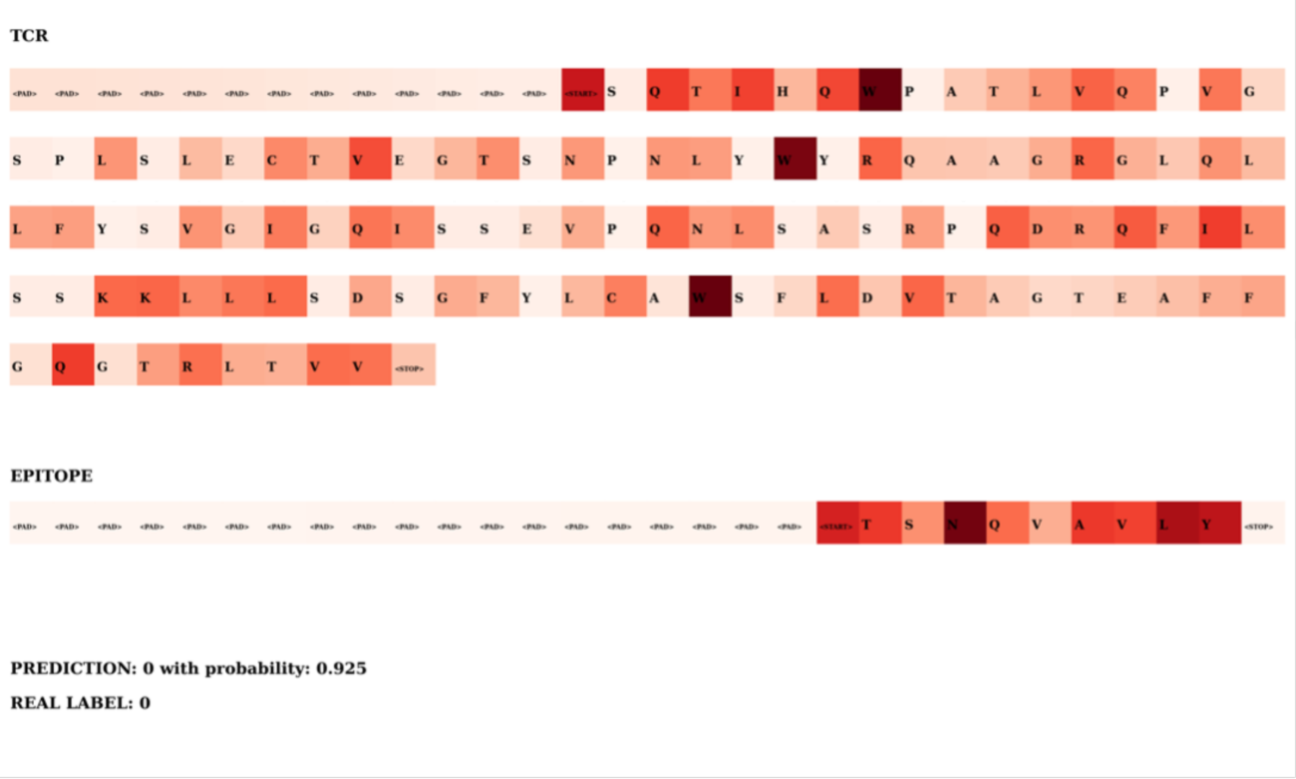

Fig. 18. FLAN importance scores after training with additional penalty to impose sparsity. The performance in the TCR-split drops to ROC-AUC: 0,80 and Balanced Accuracy: 0.,73 .

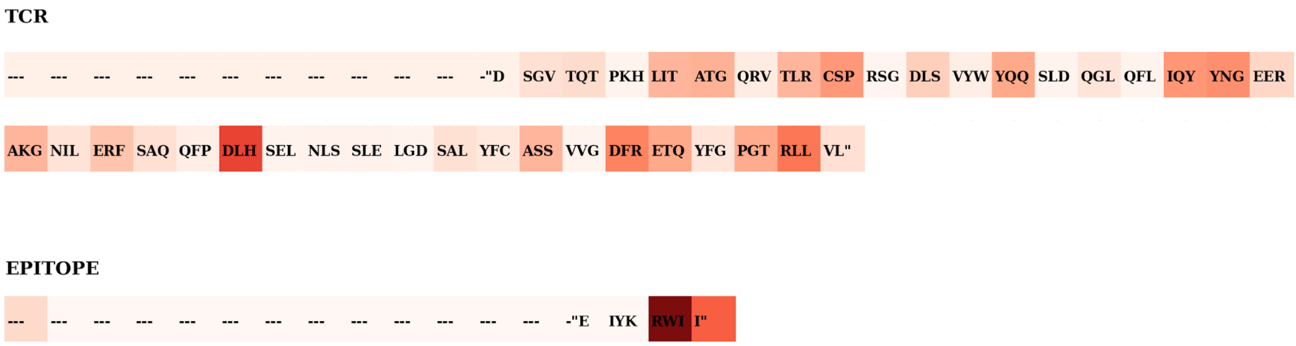

Fig. 19. FLAN importance scores for triplets of amino acids. The features are computed from non-overlapping triplets with a convolutional neural network. The performance is still comparable to TITAN [33] with ROC-AUC: 0,86/0,55 (TCR split/ strict split) and Balanced Accuracy: 0,78/0,52 (TCR split/ strict split).

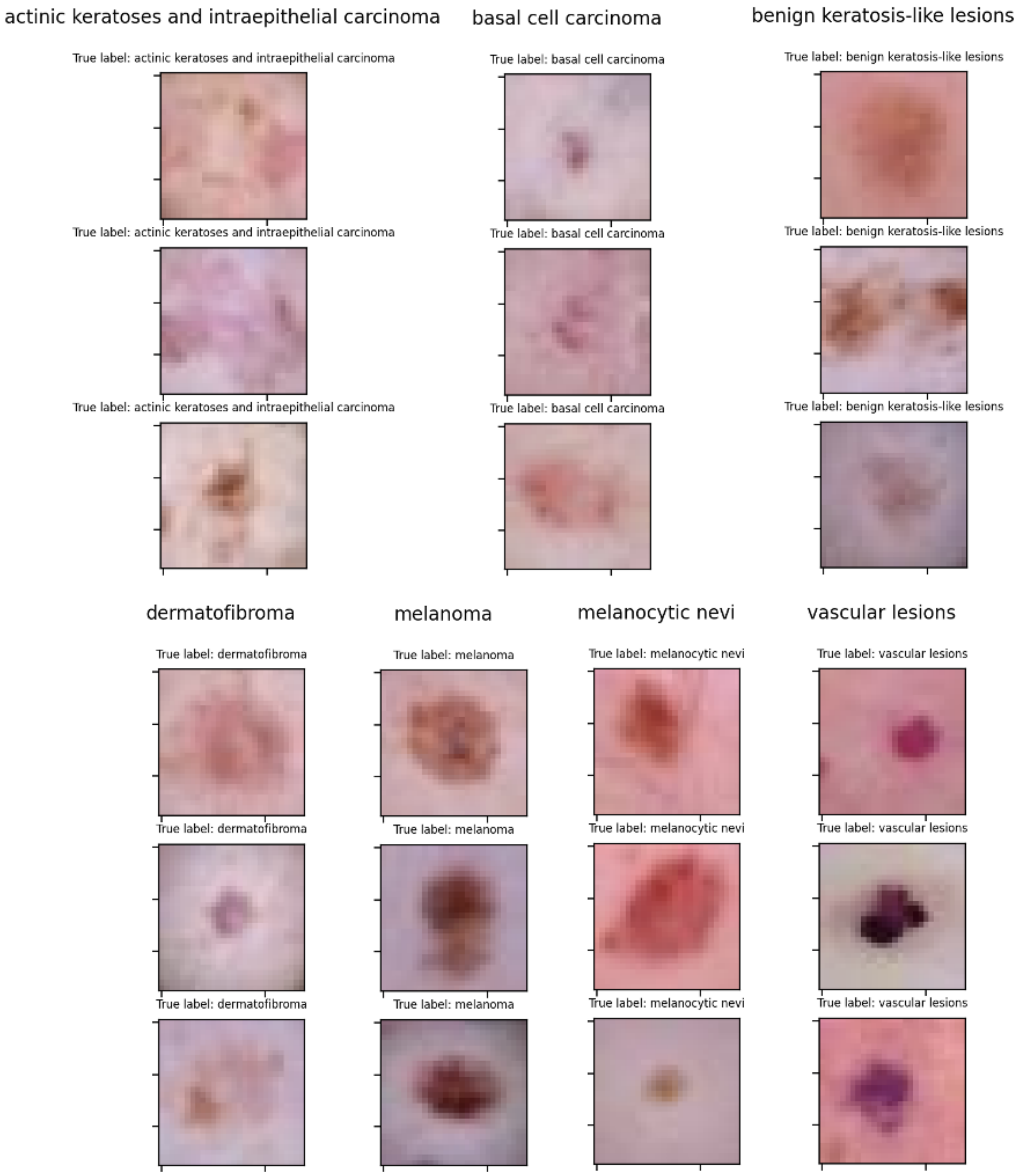

Fig. 20. The 3 most representative samples for the 7 groups. The groups are defined based on FLAN's predictions.

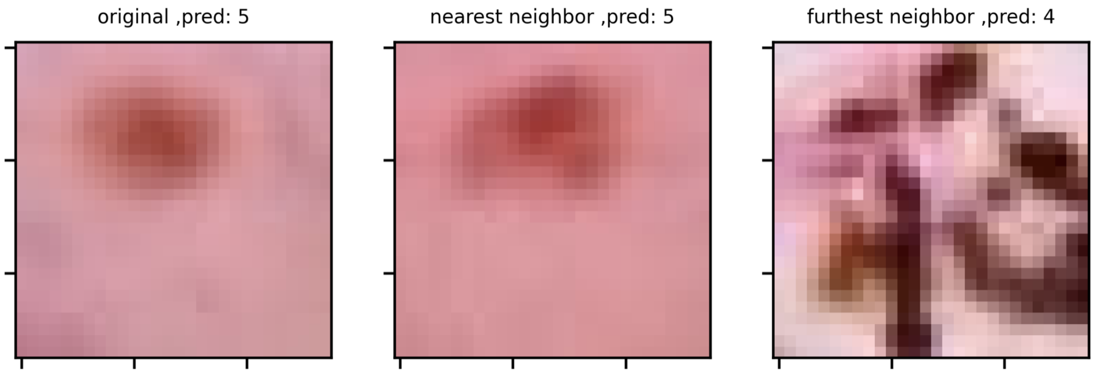

Fig. 21. The nearest and the furthest neighbor based on the sample's latent representation.

Table 9. Area Under The Curve (AUC) on Tabular Datasets.

	COMPAS	adult	heart	mammo
Logistic Regression	0.905	0.892	0.873	**0.841**
Decision Tree (small)	0.903	0.865	0.849	0.799
Decision Tree (unrestricted)	0.902	0.813	0.848	0.801
Random Forest	**0.915**	0.869	0.945	0.822
EBM [24]	0.911	**0.893**	0.941	0.840
MLP	**0.915**	0.874	0.937	0.831
SENN [13]	0.910	0.865	0.881	0.834
FLAN	0.914	0.880	**0.950**	0.832

Table 10. Test Accuracy (%) on Text Datasets.

	AGNews	IMDb
CharCNN [62]	90.49	-
LSTM [67, 68]	93.8	86.5
VDCNN [69, 70]	91.33	79.47
HAHNN [70]	-	95.17
XLNet [71]	**95.6***	**96.8***
FLAN	91.2	85.2

Additional results: non-biological datasets

Tabular Datasets

For benchmarking on tabular datasets, we follow Agarwal et al. [23] and measure the performance of different models in terms of Area Under the Curve (AUC). Table 9 shows that these datasets are easy enough that a simple logistic regression model can perform well. The results on the `adult` and `mammo` datasets suggest that linearity is a good inductive bias for these tasks, since logistic regression is able to consistently outperform all the other (non-linear) models. On the other hand, in the `heart` dataset and, to a lesser degree, in the `COMPAS` dataset, it seems beneficial to include non-linearities and interactions. In particular, FLANs closely replicates the performance of more traditional feedforward networks (MLP). This might suggest that FLANs are similar to MLPs in terms of approximation capabilities.

Text Datasets

For text datasets, we used `AGNews` [62] and `IMDb` [63]. Our model considers each token (i.e. either a word for `AGNews` and `IMDb`, or a single amino-acid for `TCR-Epitope`) in the sentence/sequence as a single feature. On the considered benchmark datasets, FLANs fair well against traditional LSTM/CNN-based models. The drop in performance of FLANs is particularly noticeable on the `IMDb` against more modern attention-based architectures. Considering that `IMDb` contains much longer sentences than `AGNews`, these results suggest that FLANs may have difficulties in learning longer term dependencies/interactions.

Image Datasets

FLANs results (Table 11) on the `MNIST` dataset are comparable to established methods. Moreover, linear models (results not reported) do not achieve more than 94% test accuracy, providing further evidence to the ability of FLANs in implementing interactions *without explicitly modeling them.* We further tested our model on the more difficult fine-grained image classification dataset `CUB-200-2011`. FLANs do not achieve the same accuracy as other models. This might be explained by the fact that the models reported are pretrained on `ImageNet` [72] and further fine-tuned on this dataset. On the other hand, in our experiments, our top-performing models use only *some layers* of a pretrained ResNeXt [73] as part of the patch feature function ϕ_i. We hypothesize that the inferior performance of FLANs is attributed to the fact that our model has to essentially *learn the interactions from scratch*, and `CUB-200-2011` might be a too small of a dataset to effectively learn this. Despite the lower accuracy, and given the relatively small size of the dataset (11.7k images split across 200 classes), we see our results as promising and a good basis for future investigations in *large scale* image recognition tasks that require interpretability.

Interpretability results

COMPAS

We use the `COMPAS` dataset as a propedeutic example to show how to interpret FLANs. We can study the approximate effect of single features separately, by applying the prediction network ψ to the feature latent representation. In this case study, this is even easier since all the features are binarized. Figure 22 shows how the predicted risk changes if we switch a feature from 0 to 1. The results suggest that the risk is *increased* for criminals that have a high number of priors, are younger than 25, are Afro-American, or have already re-offended in the past two years. Interestingly, the risk seems particularly decreased for criminals above the age of 45. To further validate these findings, we analyze the feature importances provided by our model. For each sample, we compute the importances as explained in Section 2.3.2 and then we average them over the training set. Figure 23 confirms that across the training set, the features mentioned above are the most discriminative ones. Previous analyses performed using interpretable models [13, 23] reached similar conclusions.

Table 11. Test Accuracy (%) on Image Datasets. SotA stands for State-of-the-Art, i.e. the best performing model to date.

	MNIST	SVHN	CUB
ResNet [74, 75]	99.2	94.5*	84.5*
iCaps [75]	99.2	92.0	-
ViT [76, 77]	-	88.9	90.4*
ProtoPNet [12]	-	-	84.8*
SENN [13]	99.1	-	-
SotA [78, 79]	**99.84**	**99.0***	**91.3***
FLAN	99.05	93.41	71.53**

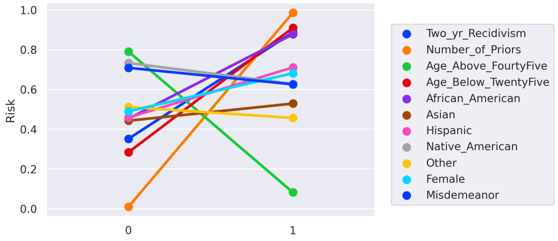

Fig. 22. Feature Effects on the COMPAS dataset

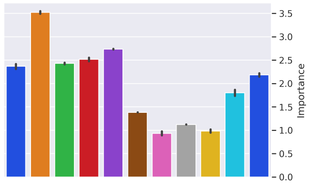

Fig. 23. Feature importances on the COMPAS dataset. Feature importances are averaged over the training set. The legend in the center shows the colors used to denote each feature.

CUB

We qualitatively validate the interpretability capabilities of our model on the more complex `CUB-200-2011` dataset. We start by comparing the feature importances natively provided by FLANs against three gradient-based *post-hoc* feature attribution methods: Integrated Gradients [18], Saliency [53], and InputXGradient [55]. Figure 24 shows the importances computed by the aforementioned methods for a test sample correctly predicted as a `Black Footed Albatross`. The norms of the latent representations (Section 2.3.2) highlight features that are typically used to identify birds, i.e. the region around the eye and the beak [12]. While noisier, gradient-based methods highlight some of the same regions, partially validating our model. However, IntegratedGradients and Saliency also highlight some areas in the top-right part of the image.

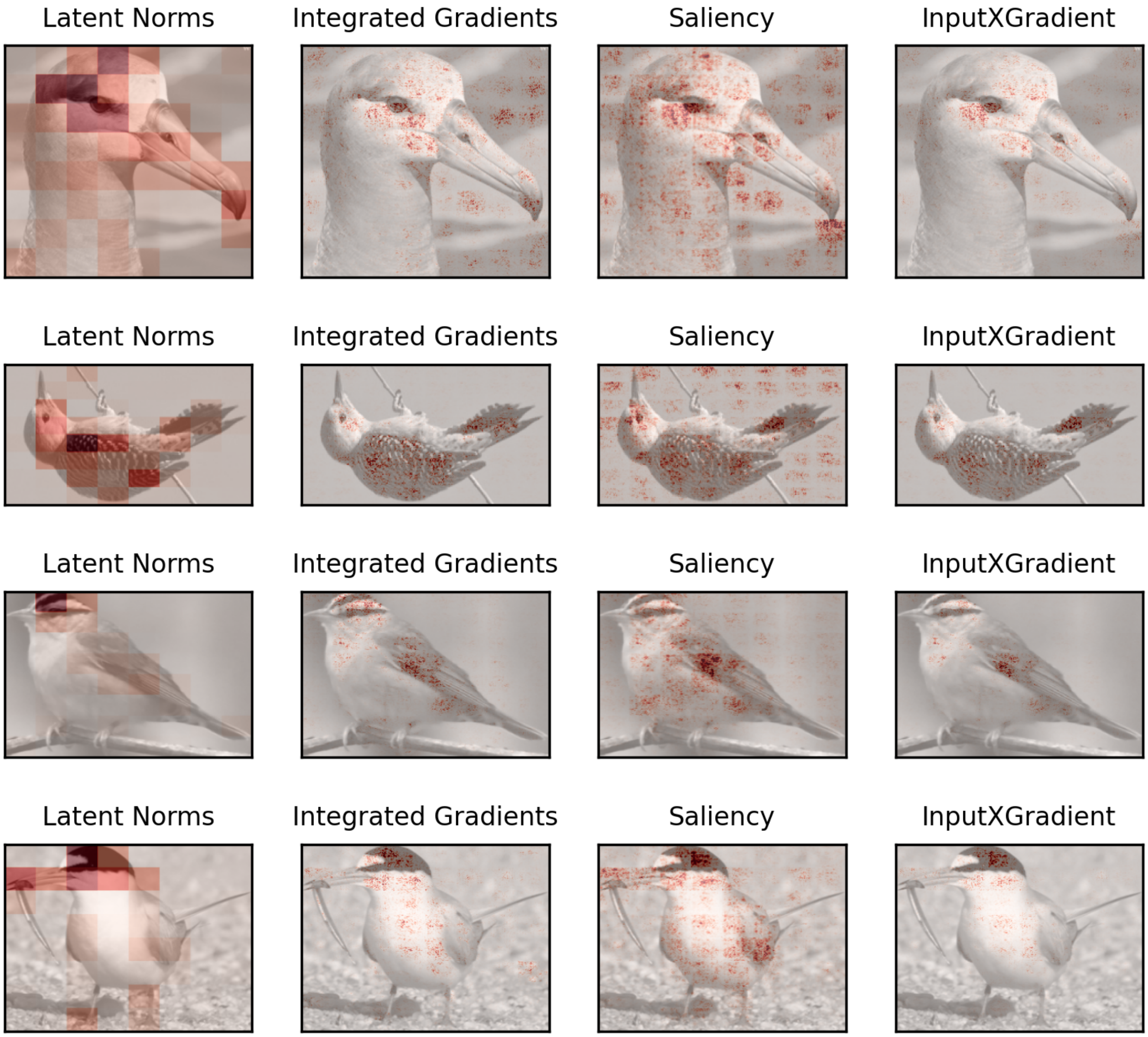

Fig. 24. Comparison of the feature importances computed by our model (Latent Norms) against 3 established gradient-based feature attribution methods in the CUB dataset.

Training details

All the deep learning models were trained using `Adam` [80] (or variants thereof, i.e. `AdamW` Loshchilov and Hutter 81, `RAdam` Liu et al. 82). Learning rates varied in the set $\{0.001, 0.0005, 0.0001, 0.00005\}$. Training was tested with no learning rate scheduling, as well as exponential decay, step decay, and cosine annealing [83] (with and without restart). The chosen hyperparameters for each experiment can be retrieved from the corresponding `config.json` file provided in the accompanying code. Details about the architectures used are also provided in the accompanying code.

For the single cells task, we used a latent space of 24 dimensions and trained the model for 100 epochs and with batch size 64. For the TCR-Epitope binding task we used 32-dim embedding vectors to encode the amino acids and then mapped them into a 128-dim latent space. We then performed 100 runs with batch size 64 (with the best set of hyperparameters). For the TCR-Epitope binding task with triplets mentioned in B.5.2 we used 32-dim embedding vectors and a convolutional NN with kernel size and stride equal to 3. We also tried different paddings to find the best set of triplets. We trained again for 100 runs with batch size 64 (with the best set of hyperparameters). For the classification of the MedMNIST dataset we used patch size and stride equal to 4 and 400 filters and 256-dim latent space. We then trained the model for 100 epochs with batch size 128 (with the best set of hyperparameters) and the scheduler proposed by Loshchilov and Hutter [84]. For the text, tabular and image datasets mentioned in C we performed 50, 10 and 5 runs with the best set of hyperparameters, respectively.
